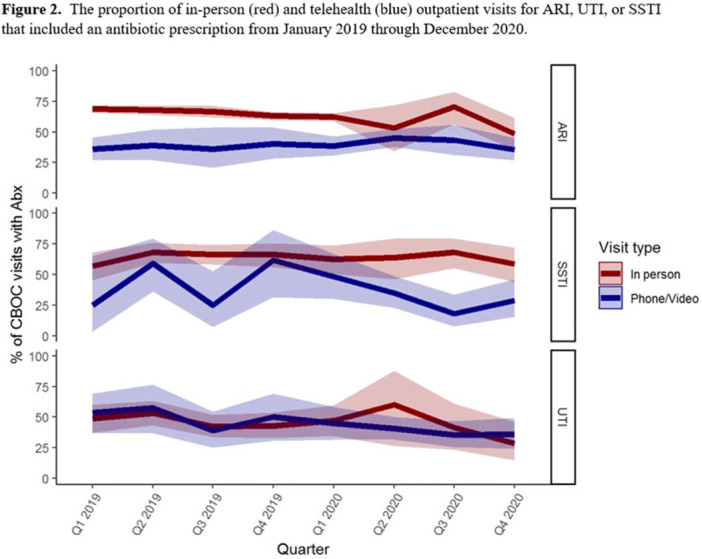# Outpatient Antibiotic Use for Common Infectious Diagnoses: Patterns in Telehealth During the Emergence of COVID-19

**DOI:** 10.1017/ash.2021.65

**Published:** 2021-07-29

**Authors:** Brigid Wilson, Taissa Bej, Sunah Song, Janet M Briggs, Richard Banks, Robin Jump, Federico Perez, Ukwen Akpoji

## Abstract

**Background:** The influence of increased use of telehealth during the emergence of COVID-19 on antibiotic prescriptions in outpatient settings is unknown. The VA Northeast Ohio Healthcare System has 13 community-based outpatient clinics (CBOCs) that provide primary and preventive care. We assessed changes in antibiotic prescriptions that occurred as care shifted from in-person to telehealth visits. **Methods:** Using VHA administrative databases, we identified all primary care CBOC visits between January 1, 2019, and December 31, 2020, that included a diagnosis for an acute respiratory infection (ARI), a urinary tract infection (UTI), or a skin or soft-tissue infection (SSTI), excluding visits with >1 of these diagnoses or with additional infectious diagnoses (eg, pneumonia, influenza). We summarized the proportion of telehealth visits and the proportion of patients prescribed antibiotics at quarterly intervals. We specifically assessed outpatient visits from April to December 2019 compared to the same months in 2020 to account for seasonality while analyzing diagnosis and antibiotic trends in the emergence of the COVID-19 pandemic. **Results:** The patients receiving care in April–December 2019 compared to April–December 2020 were similar (Table 1). From April through December 2019, 90% of CBOC primary care visits with a diagnosis for ARI, UTI, or SSTI were in-person, and antibiotics were prescribed at 63%, 46%, and 65% of visits in either modality, respectively (Figure 1). From April through December 2020, only 33% of CBOC primary care visits for ARI, UTI, and SSTI were in person, and antibiotics were prescribed at 46%, 38%, and 47% of visits in either modality, respectively. Comparing April–December in 2019 and 2020, the number of CBOC visits for ARI fell by 76% (2,152 visits to 509 visits), with a more modest decline of 20% and 35% observed for UTI and SSTI visits. In-person visits for ARIs and SSTIs were more likely than telehealth visits to result in an antibiotic prescription (Figure 2). **Conclusions:** Among the CBOCs at our healthcare system, an increase in the proportion of telehealth visits and a reduction in ARI diagnoses occurred after the emergence of COVID-19. In this setting, we observed a reduction in the proportion of visits for ARIs, UTIs, and SSTIs that included an antibiotic prescription.

**Funding:** Merck

**Disclosures:** None

**Table 1.**
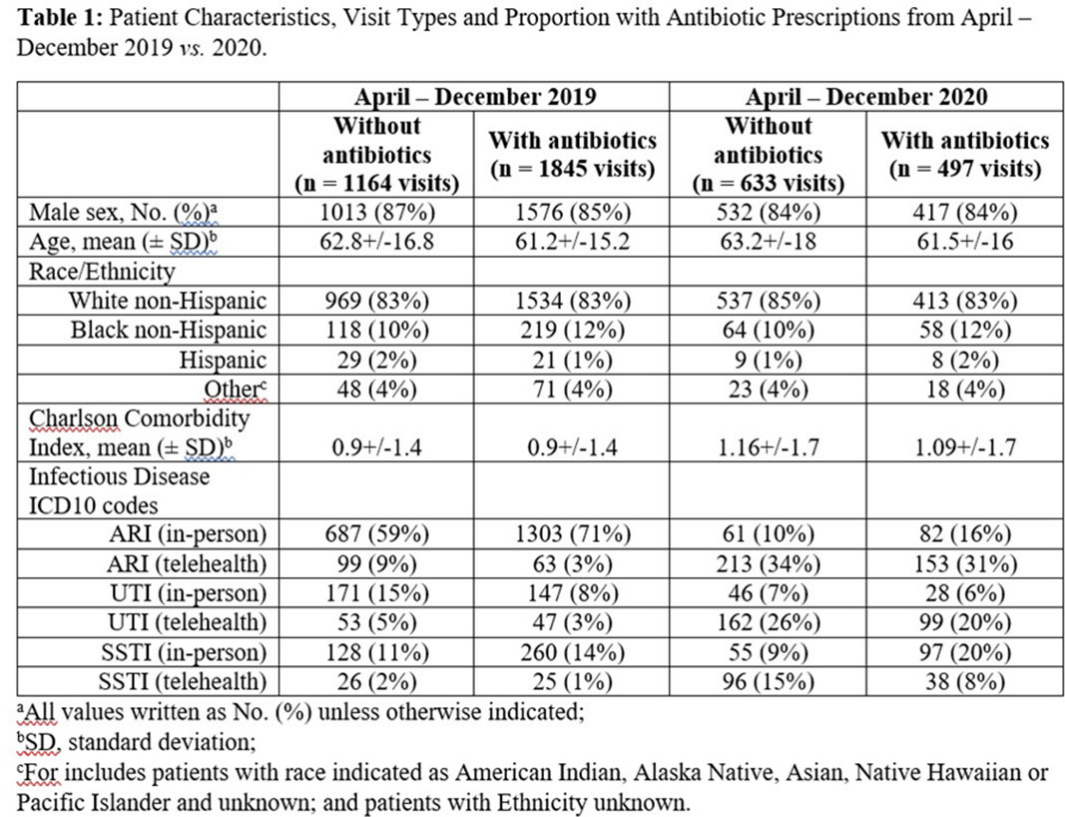

**Figure 1.**
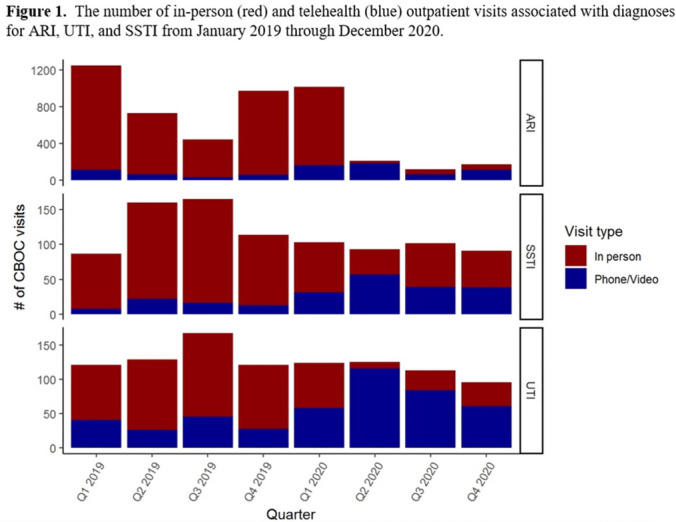

**Figure 2.**